# Coarse-graining of the dynamics seen in neural networks

**DOI:** 10.1186/1471-2202-14-S1-P115

**Published:** 2013-07-08

**Authors:** Alona Ben-Tal, Ioannis Kevrekidis, Joshua Duley

**Affiliations:** 1 Institute of Natural and Mathematical Sciences, Massey University, Auckland, New Zealand; 2 Chemical and Biological Engineering & PACM, Princeton University, Princeton, NJ, USA

## 

The pre-Bötzinger complex (pre-BötC) is a population of neurons, located in the brainstem of mammals within a central pattern generator (CPG) that produces the neural oscillation underlying the breathing rhythm. Breathing is achieved when the diaphragm (the respiratory muscle) is activated and contracts, leading to movement of air into the lungs followed by a relaxation of the diaphragm and a movement of air out of the lungs. Control of breathing is needed to maintain adequate oxygen and carbon dioxide levels in the blood and support metabolism as well as for the purpose of vocalization and is accomplished by feedback signals to the brainstem networks that affect the pre-BötC activity. Studying the integrated behavior of the respiratory system is a major challenge both computationally and conceptually, aggravated by the complex dynamics at the level of the pre-BötC population. However, the exact details of the neural dynamics may not be important when studying the integrated behavior of the respiratory system as the diaphragm contracts when excited by spiking neurons whether the spiking is chaotic or not, provided the spikes are dense enough. One would therefore like to represent the pre-BötC population with a simplified mathematical model that captures the important coarse-grained features of its dynamics.

**Figure 1:**
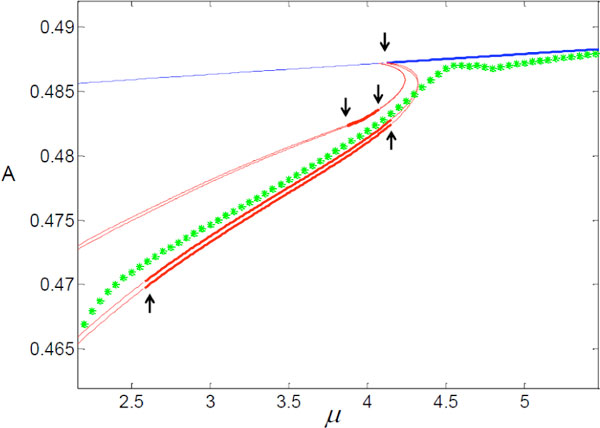
**Coarse-graining of the dynamics seen in a 2-cells neural network (described by 8 differential equations)**. A is a measure of the network activity and *μ* is a parameter related to tonic drive. The green dots represent stable equilibrium of a 2D model simplification for which the equations are unknown (calculated by our method). The blue line represents an equlibrium solution and the red lines represent periodic (spiking) solutions of the 2-cells network (in both cases, a bold line is a stable solution and a thin line is an unstable solution). The arrows indicate bifurcation points in the 2-cells network; these bifurcations are not captured by the simplified representation. The solutions of the 2-cells network were calculated by AUTO.

Motivated by the example above, we have developed a numerical method that enables us to estimate the dynamics a simplified model should retain. The method maps between the variables of a bursting neural network (for which the equations are known) and the variables of a simplified model (for which the equations are unknown). By moving backward and forward between the variables of the detailed neural network and the variables of the simplified system using restriction and lifting operators, and simulating the detailed neural network for short periods of time, one can calculate the steady states of the simplified model and their stabilities and create bifurcation diagrams for the simplified model even though the equations of the simplified model are unknown. Using this approach we created bifurcation diagrams for 2D simplified models representing networks of 1 to 50 cells and compared them with bifurcation diagrams of the detailed networks (see Figure 1 for an example). Our calculations show that a 2D model can capture the dynamical behavior of the neural system roughly for certain parameter values and identify the domain of parameters for which the 2D simplification is invalid.

